# The Role of Intraoperative Functional Lumen Imaging in Peroral Endoscopic Myotomy and Laparoscopic Heller Myotomy

**DOI:** 10.1016/j.atssr.2025.05.004

**Published:** 2025-05-28

**Authors:** Jessica M. Carducci, Andrew C. Chang, Kiran H. Lagisetty, Richard Kwon, Jules Lin, Rishindra M. Reddy

**Affiliations:** 1 University of Michigan Medical School, Ann Arbor, Michigan; 2 Section of Thoracic Surgery, Department of Surgery, Michigan Medicine, Ann Arbor, Michigan; 3 Division of Gastroenterology, Department of Internal Medicine, Michigan Medicine, Ann Arbor, Michigan

## Abstract

**Background:**

Peroral endoscopic myotomy (POEM) and laparoscopic Heller myotomy (LHM) are 2 common treatments for achalasia. Endoluminal functional lumen imaging probe (EndoFLIP; Medtronic) technology allows for real-time esophagogastric junction measurements intraoperatively before and after myotomy. We hypothesize that POEM would result in improved distensibility over LHM given its longer myotomy length.

**Methods:**

A retrospective cohort study of prospectively collected data from consecutive patients with achalasia who underwent either POEM or LHM at a single-center tertiary care center between 2018 and 2019 was performed. Premyotomy and postmyotomy EndoFLIP measurements and outcomes such as length of stay and 30-day readmissions were compared using χ^2^ and *t* tests.

**Results:**

Data from 65 patients with achalasia were included (19 LHM and 46 POEM), and 57 patients were excluded for lack of EndoFLIP data. The POEM cohort was significantly older (*P* < .001), with no other significant demographic differences. Average pre-LHM and post-LHM distensibility (1.22 [SD 0.5] and 3.64 [1.14], respectively) represented a mean 198% ( 41%) increase. Pre-POEM and post-POEM distensibility measurements (1.73 [1.41] and 4.1 [2], respectively), demonstrated a mean 137% (39%) increase (*P* = .95). The esophagogastric junction diameter increased by 73% (42%) and 49% (41%) in LHM and POEM, respectively (*P* = .07). There was no significant difference in length of stay (*P* = .06) or 30-day readmissions (*P* = .17).

**Conclusions:**

Similar increases in EndoFLIP measurements were seen intraoperatively during POEM and LHM. Longer-term quality of life data should be used to correlate with EndoFLIP measurements to understand the benefits of myotomy.

**In Short**

▪ Measurement of preprocedural and postprocedural distensibility during myotomy allows for real-time feedback of myotomy efficacy and provides quantitative guidance for treatment decisions intraoperatively.
▪ POEM and LHM demonstrate similar increases in EGJ distensibility.
▪ Longer-term quality of life data and gastroesophageal reflux reporting should be used to correlate with EndoFLIP measurements to understand the benefits of the different types of myotomy.

Achalasia is an esophageal motility disorder characterized by degeneration of the myenteric plexus at the esophagogastric junction (EGJ) and resulting in impaired relaxation of the EGJ as well as impaired esophageal peristalsis leading to regurgitation, dysphagia, and chest pain.^1^, ^2^, ^3^ The retention of food can lead to chronic esophageal inflammation, increasing patients’ risk of esophageal cancer and severely limiting their quality of life.^4^ The exact cause of the disease remains unknown, although it is hypothesized that genetically susceptible individuals are at higher risk for viruses that trigger inflammatory and autoimmune responses toward the inhibitory neurons at the EGJ.^1^ Achalasia is more common than previously thought. A 2022 study suggested 2- to 3-fold higher rates of achalasia in the United States, with a prevalence and annual incidence of 162.1 per 100,000 and 26.0 per 100,000, respectively.^2^

Currently, the degeneration of the myenteric plexus is considered irreversible, and treatment options for achalasia are deemed palliative. The primary aim is often symptom relief, which is usually measured by a reduction in a patient’s Eckardt score.^3^ Transthoracic esophagogastric myotomy was the initial surgical option developed in 1913.^5^ With the advent of laparoscopy, laparoscopic esophagogastric myotomy has become the most common surgical method to treat achalasia.^3^ The introduction of peroral endoscopic myotomy (POEM) provided a novel alternative to the traditional surgical approach and has been increasingly used in practice since 2010.^4^ Until POEM was developed, laparoscopic Heller myotomy (LHM) with fundoplication (to act as an antireflux barrier) was considered the “gold standard” surgical treatment of achalasia.^5^ However, both forms of myotomy have recently demonstrated similar benefits with no difference in dysphagia at 5 years.^5^ Other studies have suggested that the lack of an antireflux procedure after POEM has led to higher rates of silent reflux compared with LHM.^5^ Longer-term outcomes are still under investigation, and therefore the best treatment option remains controversial. LHM usually involves a 4- to 6-cm myotomy, whereas POEM involves a longer (8- to 10-cm) myotomy.

The emergence of endoluminal functional lumen imaging probe (EndoFLIP) technology has allowed physicians to assess the results of their myotomy at the time of intervention quantitatively.^6^ EndoFLIP permits measurements of the mechanical properties of the esophagus and EGJ during volume-controlled distention.^7^ Its role in achalasia is evolving because EndoFLIP provides additional and complementary information to traditional manometry in the diagnostic phase for achalasia as well.^7^ Measuring the distensibility of the EGJ, which is largely responsible for esophageal emptying and is correlated with symptom severity,^7^ has been proven to be better than measuring lower esophageal sphincter pressure for evaluating treatment efficacy.^7^ Measurement of preprocedural and postprocedural distensibility during myotomy allows for real-time feedback of myotomy efficacy and provides quantitative guidance for treatment decisions intraoperatively. Because our institution (Michigan Medicine, Ann Arbor, MI) has developed a POEM program, we hypothesized that POEM would demonstrate superior postmyotomy EGJ distensibility as measured by EndoFLIP over LHM, given its longer myotomy length.

## Patients and Methods

A retrospective review of prospectively collected data from consecutive patients with achalasia who underwent either POEM or LHM with concurrent EndoFLIP measurements at a single-center tertiary center between January 1, 2018 and September 31, 2019 was performed. Patients were excluded from analysis if there was no EndoFLIP procedure report available. Patients were identified using an established surgical database. LHM was performed by 5 different general thoracic surgeons, whereas POEM was performed by a single general thoracic surgeon working with a single gastroenterologist. All LHM operations included an antireflux operation (anterior [Dor] fundoplasty). In general, POEM myotomy length was 8 cm, although it was extended to 10 cm for patients with type III achalasia. LHM myotomy length was 6 cm, 4 cm above the GEJ and 2 cm below it. EndoFLIP measurements were performed before and after the myotomy and before the fundoplasty (for LHM). Myotomies were extended as needed using EndoFLIP measurements and clinical input from the endoscopy on the basis of surgeon experience. The goal was to achieve distensibility within the 3 to 4 cross-sectional area (CSA, mm^2^)/intraballoon pressure (mm Hg) range. The decision about which type of approach varied by referral mechanism, patient preference, and insurance coverage. The postoperative recovery pathway for all patients initially included an overnight stay with an esophagogram on postoperative day 1, followed by discharge on a liquid diet. For patients who underwent POEM, this shifted to a same-day discharge at the end of the study period.

Medical records were reviewed for patient demographics, clinical characteristics (including EndoFLIP measurements), and outcomes such as length of stay (LOS), readmissions, and surgical complications. Premyotomy and postmyotomy EndoFLIP measurements included diameter (mm) and distensibility (CSA [mm^2^]/intraballoon pressure [mm Hg]). All analyzed measurements were taken with the 325-NF model (Medtronic) at the same catheter size (8 cm) and balloon inflation (40 mL). Data for all continuous variables were expressed as mean (SD). Categorical variables were analyzed and compared using the χ^2^ test or the Fisher exact test. Continuous variables were analyzed using 2-sided *t* tests. Statistical significance was set at *P* <.05. This study was approved by our Institutional Review Board (HUM00047546).

## Results

A total of 65 patients underwent esophagomyotomy with EndoFLIP measurements during the study period, including 19 patients who underwent LHM and 46 patients who underwent POEM. The average age was 57.66 (SD 17.31) years, 57% were male, and 88% were White. The POEM cohort was significantly older (*P* < .001), with no other significant demographic differences (Table). No procedure-related complications occurred in either group, including return to operating room, unexpected intensive care unit admission, and any pulmonary, cardiac, or neurologic events. There were two 30-day readmissions in the POEM group (for syncope and nausea, vomiting, and diarrhea) and none in the LHM group. No significant difference was noted in LOS between the groups (*P* = .06) although the LHM group trended to have a longer stay (1.37 [0.83] days vs 0.97 [0.37] days, respectively).

**Table:** Baseline Characteristics of Patients Who Underwent Laparoscopic Heller Myotomy or Peroral Endoscopic Myotomy

Characteristics	LHM (n = 19)	POEM (n = 46)	Total (n = 65)	*P* Value
Age, y	45.89 [15.13]	62.51 [15.87]	57.66 [17.31]	**<.001**
Male	56.25	63.04	56.92	.84
White	84.21	89.13	87.69	.58
Body mass index, kg/m^2^	31.04 [7.26]	29.68 [7.74]	30.08 [7.57]	.51
Weight loss in past 3 mo, kg	0.88 [1.33]	0.68 [1.25]	0.72 [1.26]	.59

Values are mean (SD) or %. Age was the only demographic that was statistically significant between groups, which is represented in bold.

LHM, laparoscopic heller myotomy; POEM, peroral endoscopic myotomy.

**Figure 1:**
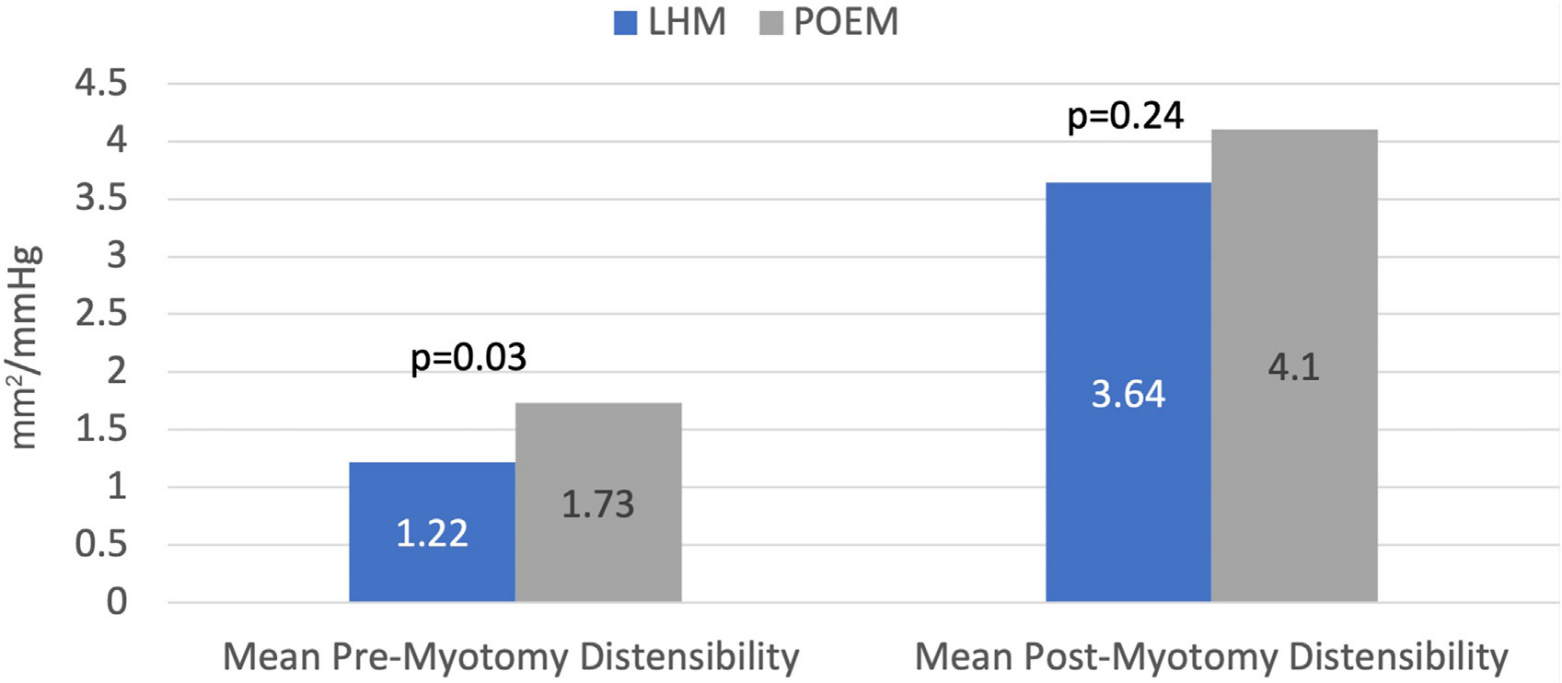
Differences in premyotomy and postmyotomy esophagogastric junction distensibility. (LHM, laparoscopic Heller myotomy; POEM, peroral endoscopic myotomy.)

**Figure 2:**
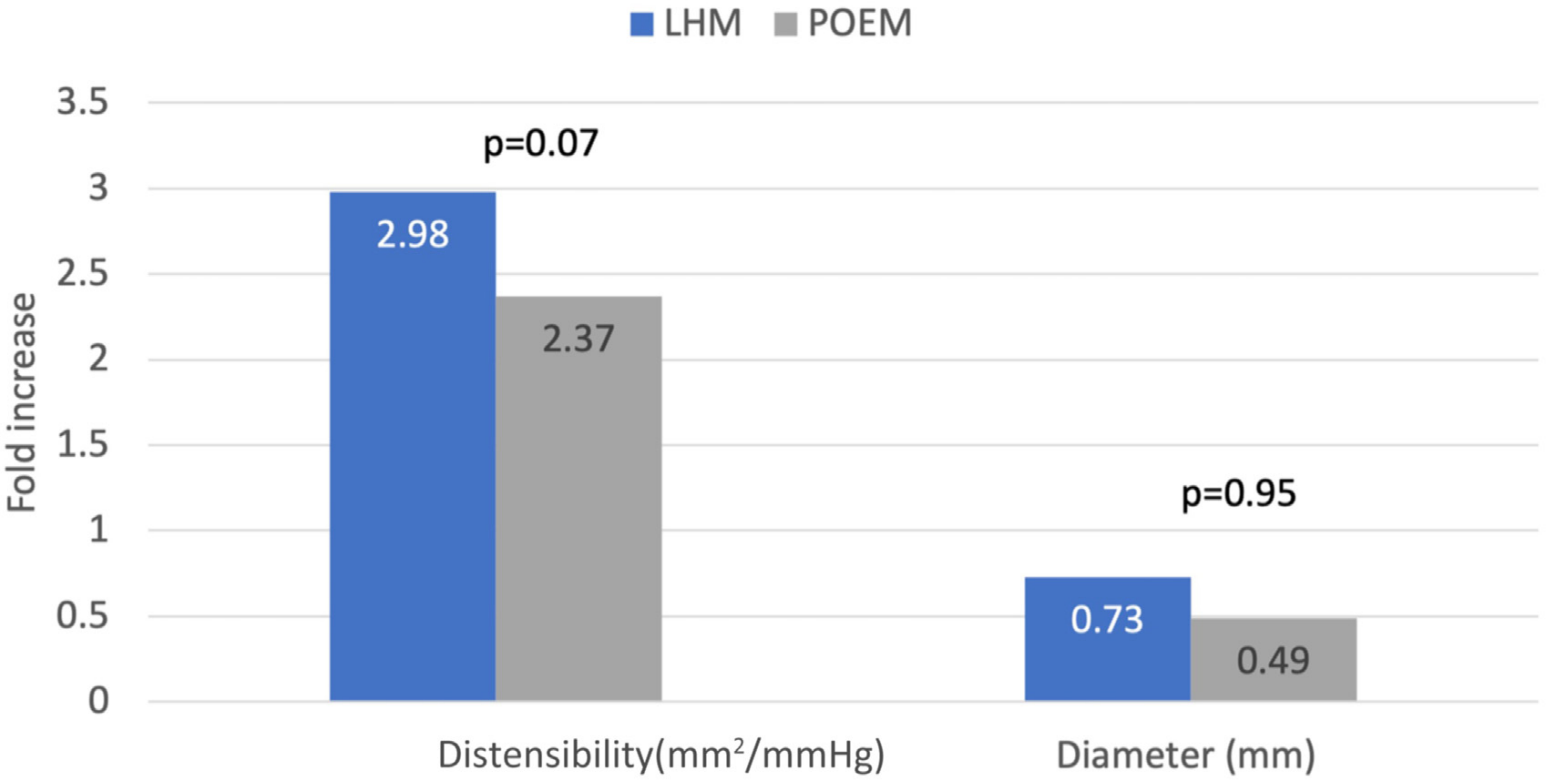
Average fold increase in endoluminal functional lumen imaging probe EndoFLIP measurements. (LHM, laparoscopic Heller myotomy; POEM, peroral endoscopic myotomy.)

**Figure 3:**
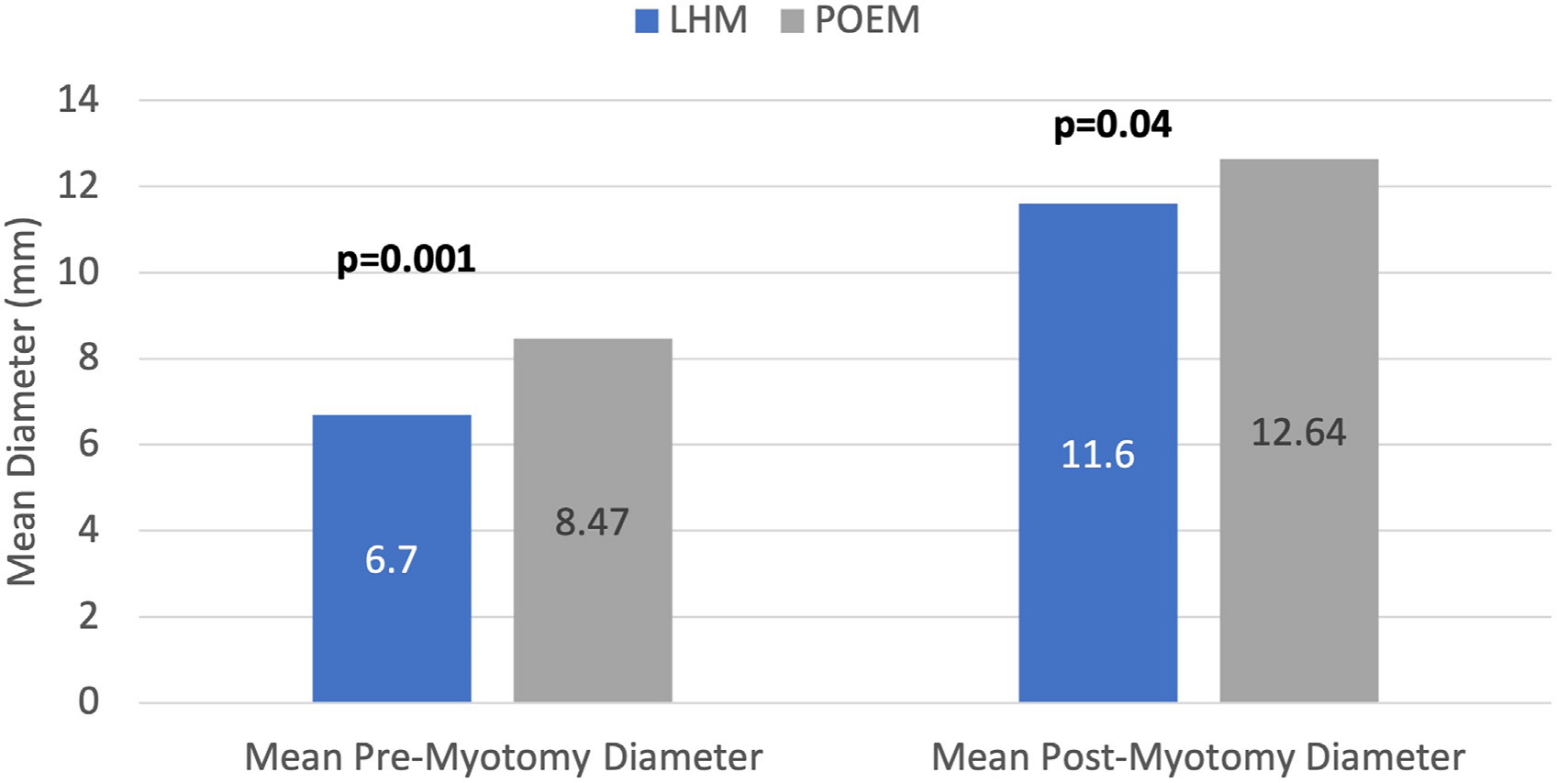
Differences in premyotomy and postmyotomy esophagogastric junction diameter. (LHM, laparoscopic Heller myotomy; POEM, peroral endoscopic myotomy.)

The average pre-LHM and post-LHM distensibility (Figure 1) was 1.22 (0.5) and 3.64 (1.14), respectively, representing a mean 2.98 (1.41)–fold increase. Distensibility before and after POEM (1.73 [1.41] and 4.1 [2], respectively; *P* = .95) increased by a mean 137% (1.39%)–fold (Figure 2). Additionally, EGJ diameter had a moderate postprocedure increase (Figure 3), with slightly more expansion observed in LHM (1.73 [0.42]) than POEM (1.49 [0.41]), although the statistical difference between the 2 methods was not statistically significant (*P* = .07).

## Comment

The role of EndoFLIP in the diagnosis and evaluation of achalasia has been extensively described in the literature; however, this technology is not consistently used in the operating room. Not only does EndoFLIP allow direct measurement of EGJ distensibility, CSA, and intraluminal pressure during volume-controlled distention,^7^ but also surgeons can assess the effectiveness of their myotomy in real time. The ability to assess and then augment the myotomy intraoperatively allows more precise prediction of the myotomy’s effects on EGJ function and minimizes the risk of residual dysphagia or the development of GERD.^6^

Our results are similar to those of an initial study by Teitelbaum and colleagues,^8^ who evaluated 11 patients who underwent LHM and 14 patients who underwent POEM with an intraoperative EndoFLIP study and found no difference in EGJ distensibility between the groups. However, these investigators performed a second study^9^ analyzing 20 patients who underwent LHM and 36 patients who underwent POEM and found that both groups demonstrated an increased distensibility index (defined as minimum CSA divided by intraballoon pressure), although this increase was significantly larger with POEM (7 [3.1] vs 5.1 [3.4] mm^2^/mm Hg; *P* <.05). Although both of our groups also demonstrated an increase in distensibility, the average fold increase was similar for both groups. Teitelbaum and colleagues^9^ noted improved technique in POEM as a possible explanation for the difference between their 2 studies. Our data do not capture whether additional tissue dissection or extension of an initial myotomy was performed for any given patient. In addition, this study reflects an early period of POEM adoption in our institution, with outcomes possibly related to the learning curve for this novel approach.

It is also important to consider standardization of approaches. Su and colleagues^10^ presented an expert consensus on the intraoperative use of EndoFLIP and provided several best practices to consider. These investigators noted that attention to the intraballoon pressure (mm Hg) at the maximum diameter of the narrowest luminal area is important. The accuracy of distensibility measurements relies on ensuring that the intraballoon pressure is >15 mm Hg to allow adequate luminal distention. Measurements taken at 40-mL volume are encouraged because this amount ensures that intraballoon pressure is always >15 mm Hg. Although our measurements were all taken at the same catheter size (8 cm) and balloon volume (40 mL), variability could still exist in users’ calibration, placement, interpretation of real-time data, and subsequent intraoperative adjustments to surgical technique. In addition, some ambiguity exists in determining the spastic segment length to tailor the myotomy in type III achalasia. A myotomy length of 10 cm is performed at our center, but other centers prefer 16 cm, and there is limited research to support any 1 method. Therefore, development of standardized protocols and guidelines for the procedure’s integration into surgical workflow are needed to achieve consistent and reliable EndoFLIP measurements.^10^

This study has several limitations, mostly resulting from its retrospective design and limited number of patients. This was not a randomized cohort, and only 1 surgeon performed both POEM and LHM. This study’s low number of patients limits the statistical impact of the results. Some patients who preferred POEM did not have insurance coverage and underwent LHM. The captured cohort reflects only patients with documented EndoFLIP measurements. The trend toward a shorter LOS in the POEM cohort was likely the result of a switch to this procedure’s being performed on an outpatient basis with same-day discharge during the study period. The older age of the POEM cohort could possibly reflect perceived surgical risk.

In conclusion, when considering LHM or POEM, our findings provide objective data collected by EndoFLIP that show similar increases in EGJ measurements. Average distensibility increased by approximately 3-fold after LHM and by roughly 2.4-fold after POEM, both with considerable variation among patients, although not achieving significance. Longer-term quality of life data and gastroesophageal reflux reporting should be used to correlate with EndoFLIP measurements to understand the benefits of the different types of myotomy.
